# Is Hip Muscle Flexibility Associated with Low Back Pain Among First-Year Undergraduate Students?

**DOI:** 10.3390/jcm13247598

**Published:** 2024-12-13

**Authors:** Janan Abbas, Noa Reif, Kamal Hamoud

**Affiliations:** Department of Physical Therapy, Zefat Academic College, Zefat 13206, Israel; noa.re@zefat.ac.il (N.R.); kamalh@zefat.ac.il (K.H.)

**Keywords:** low back pain, muscle flexibility, physical activities, students

## Abstract

**Background/Objectives:** Hip muscle lengthening is commonly associated with the normal function of the lumbar spine and lower extremities. Some evidence correlates hamstring and iliopsoas tightness with low back pain (LBP). Undergraduates are more prone to LBP as they are involved in prolonged sitting and poor posture. This study aims to assess the impact of hip muscle lengthening on LBP. **Methods:** This article involves a descriptive study of 70 students who were recruited from Zefat Academic College. Measurement of hamstrings and iliopsoas muscle lengthening, as well as a constructive questionnaire, were used. **Results:** The majority of participants (80% for hamstrings and 96% for iliopsoas) manifested normal muscle lengthening. Muscle flexibility was significantly higher among females. Logistic regression analyses revealed that hamstring lengthening (right) and stress-related study are significantly associated with LBP. **Conclusions:** The current study indicates that muscle length is female-dependent and right–left muscle length is symmetrical. Increased hamstring length could be related to LBP.

## 1. Introduction

Flexibility of muscles is considered an essential element of normal biomechanical function [1] and optimizing the performance of physical activities [2]. Reduced flexibility not only decreases the range of motion but can also lead to various other musculoskeletal problems [3]. It is well established that the sedentary behavior of developed nations could augment muscle dysfunction and lead to musculoskeletal discomfort [4,5]. In addition, poor posture due to imbalanced muscles and decreased flexibility is considered a risk factor for back pain [6,7]. 

There is a plethora of evidence supporting the assessment and treatment of the range of motion of the hip joint in individuals with chronic low back pain [8,9,10,11,12].

Hip muscles such as the hamstrings are commonly linked with movement dysfunction at the lumbar spine complex and lower extremities, and have been coupled with low back pain [13]. Evidence has also correlated a decrease in psoas lengthening to LBP due to the connection of this muscle to the pelvis and lumbar spine [14]. It has been postulated that psoas tightness may lead to lumbar hyperlordosis, predisposing to apophyseal facet impingement, which may produce pain in the low back [15]. In addition, hamstring tightness was found to have a positive correlation with the severity of LBP [16]. 

Low back pain (LBP) is a widespread health problem that affects individuals of all ages and professions. It is one of the most common musculoskeletal disorders worldwide [17], leading to disability and economic burdens. Moreover, LBP profoundly impacts quality of life, productivity, and mental health [17,18]. Etiologically, LBP is caused by several independent factors [19] and involves various structures such as facet joints, intervertebral discs, and muscles. Although LBP increases in the elderly, its prevalence among adolescents (18–24 years) is higher (up to 40%) [20,21]. Undergraduate students, particularly in the health sciences, are at high risk for developing low back pain due to the demanding nature of the curriculum, physical exposure at clinical practice, and prolonged sitting [22,23,24]. Despite the importance of hip muscle flexibility for maintaining joint mobility and relieving musculoskeletal disorders [8,9,10,11,12], few studies have addressed the impact of these muscles on LBP. Additionally, the existing data regarding hip muscle flexibility and LBP are ambiguous. For example, two recent systematic reviews displayed inconsistent findings regarding hip muscle lengthening and LBP [25,26]. 

Hence, this study aims to characterize hip muscle flexibility among first-year undergraduate students and to reveal whether hip muscle lengthening is associated with LBP. 

## 2. Materials and Methods

**Figure 1:**
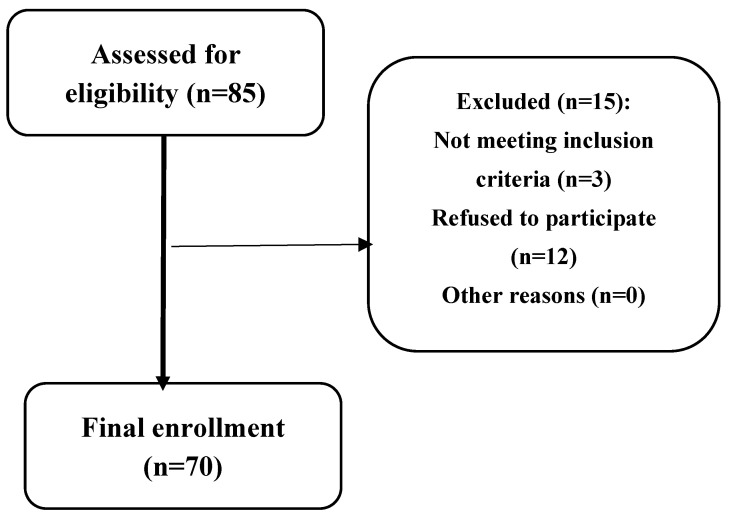
Flow chart for the participants’ assignment and enrollment.

A descriptive study was conducted in the 2023 and 2024 academic years at Zefat Academic College in the north of Israel. We adopted different channels for recruiting participants to ensure a diverse sample. This included advertisements posted in the local academic institution’s social media, local department forums, and the college’s website. Out of the 85 first-year students who responded to the research invitation, 70 met the inclusion criteria and agreed to participate for this study [Figure 1]. Participants were excluded if they were (1) pregnant, (2) underwent surgery in their spine or lower extremities, (3) had neuromuscular-skeletal diseases, and/or (4) had anatomical deformities related to the spine and chest wall. A consent form, which included the purpose of the research and the right of the participant to withdraw at any time, was received from each participant. We recruited only first-year students because this research is part of a prospective study that intends to follow up with the students for 1–2 years. This study was conducted according to the Helsinki Declaration and approved by the Departmental Research Ethics Committee, Zefat Academic College (no. 2-2024). 

### 2.1. Instruments and Measurements 

Structured questionnaire. We used a modified validated Standardised Nordic Questionnaire [27] that sought information on sociodemographic characteristics, physical activities, and factors related to sedentary behavior and smoking habits [28,29]. Students were also asked if they had suffered from LBP in the last week (for at least 12 h). Pain intensity was measured following the visual analog scale (VAS) [30]. LBP was defined as an ache or discomfort localized below the costal margin and above the inferior gluteal folds with and without leg pain. 

Data about study-related stress were also recorded [28]. In this matter, the students had to classify their feelings of stress during the last month as (a) very high to quite high or (b) little to none. 

Muscle flexibility. Hamstring and iliopsoas length was evaluated through passive straight-leg raise [31] and the modified Thomas test [32], respectively. 

Muscle length measurement. Hamstring length measurement was conducted with participants in a supine position considering a stabilization of the contralateral thigh. The hip was passively flexed (maintaining knee extension and relaxed ankle) to the limit of motion, in which the examiner’s perception was stretch-firm and the participant felt a strong and tolerable stretch without soreness. The goniometer axis was placed over the participant’s femur’s greater trochanter as the stationary and movable arms were parallel to the trunk line and the longitudinal axis of the femur (pointing toward the lateral epicondyle), respectively. A modified Thomas test was carried out with the participant sat close to the examination table. The participant was then instructed to pull their uninvolved knee toward their chest to the point that the lumbar lordosis was flattened. With the assistance of the examiner, the participant slowly rolled backward onto the table. The involved limb was then allowed to hang unsupported off the table, preventing abduction of the hip. The examiner palpated the lumbar spine to confirm that it remained in contact with the table. When the final test position was achieved, the examiner placed the fulcrum of the goniometer over the lateral aspect of the greater trochanter. The proximal arm was aligned with the lateral midline of the pelvis, and the distal arm was aligned with the lateral midline of the femur using the lateral epicondyle for reference.

One of the authors (NR), who was unaware whether the participant suffered from LBP, measured the muscle length on both sides. Each side was evaluated three times and the mean value was then recorded. Hamstring tightness was defined when the range of passive hip flexion was less than 80 degrees, whereas the inability of the hip to extend to a neutral position was considered iliopsoas tightness [33].

Body anthropometry. One of the authors (NR) evaluated each participant for height and weight using a digital device (Shekel, H150-5). The BMI value was calculated as the weight (kg) divided by height in meters squared (m^2^). 

### 2.2. Statistical Analyses

The data were analyzed using the SPSS software 25. The intra-year correlation coefficient was calculated to determine the intra-tester and inter-tester reliability of the measurements of muscle flexibility within 5 to 7 day intervals (repeated measurements of 15 individuals). All continuous parameters (e.g., age, weight, and muscle length) were checked for normal distribution when running the Kolmogorov–Smirnov test. An independent *t*-test was used to check the association between muscle length and LBP, gender, and physical activities. Paired *t*-test was carried out to reveal the right–left muscle length asymmetry. Logistic regression analysis (backward LR) determined the variables associated with LBP (dependent variable—LBP, independent variables—age, BMI, muscle length, prolonged sitting, etc.). A significant difference was set at *p* < 0.05. 

## 3. Results

The intra-tester and inter-tester reliability results (ICCs) for measuring the hamstring and iliopsoas lengthening were very high: 0.995 to 0.985 and 0.992 to 0.942, respectively. 

The demographic and sedentary features of the participants are presented in Table 1. From the total sample of 70, we enrolled 13 males (19%) and 57 females (81%). The study sample’s mean age and BMI values were 25 ± 6 years and 23.7 ± 4, respectively. About 81% of the participants were female, and 11% were habitual smokers. Forty-six percent of the students suffered from LBP, 27% had chronic diseases, and about half (56%) experienced high levels of stress (very high to quite high). In addition, half of the participants were involved in physical activities: 50% for aerobic training and 46% for anaerobic activity. Forty-nine percent of students spent time in a prolonged sedentary position (>3 h). 

### 3.1. Muscle Length Characteristics

The majority of the participants had a normal muscle length (80% for the hamstrings and 96% for the iliopsoas) (Table 1). Females manifested greater muscle flexibility for the hamstrings and iliopsoas than males, adapted for the same age and BMI (*p* < 0.05) (Table 2). In addition, individuals who were engaged in anaerobic physical activity (e.g., yoga) revealed significant muscle flexibility compared to those who did not practice this activity (*p* < 0.05). A high correlation was reported between the flexibility of the hamstrings and iliopsoas muscles (r = 0.692; *p* < 0.001). In contrast, no significant asymmetry was noted for hip muscle lengthening (hamstrings right–left: 92.1 ± 17 vs. 91.8 ± 17; *p* = 0.734, and iliopsoas right–left: 11.5 ± 8 vs. 12.4 ± 7; *p* = 0.08). Notably, this trend was also established when the analysis was conducted separately for individuals with and without LBP. 

### 3.2. Muscle Flexibility and LBP 

No significant difference was found in the mean age of participants between the group with LBP and the group without LBP (25 ± 5 vs. 24.5 ± 4, *p* = 0.795). Participants with LBP displayed greater hip muscle flexibility than those without LBP; however, significant differences were noted only for the hamstring muscles (Table 3). The logistic regression analyses showed that right hamstring length (OR = 1.035, *p* = 0.033) and study-related stress (OR = 3.836. *p* = 0.013) increase the likelihood of LBP among undergraduate students (Table 4).

## 4. Discussion

The results of this study indicate that hamstring flexibility (OR = 1.035) and study-related stress (OR = 3.836) increased the risk of LBP among first-year undergraduates. In addition, females manifested more muscle (hamstrings and iliopsoas) length than males. 

The association between hip muscle flexibility and LBP is in agreement with many previous studies [34,35] However, it is not apparent whether muscle tightness or increased muscle flexibility could lead to LBP. For example, Noormohammadpour and colleagues previously reported that adolescent girls who had greater spinal forward bending and increased hip joint range of motion (ROM) for internal rotation were factors associated with LBP [36]. Partial support could also be attained from some evidence [36,37,38,39] which reported that joint hypermobility was related to LBP. Indeed, joint hypermobility was not examined in the current study, and no cut-off for the extreme range of hamstring flexibility was recorded. Yet, the association between increased hamstring flexibility and LBP could be attributed to the fact that augmentation of the joint range of motion could lead to soft tissue strain and wearing of the joint surfaces. On the contrary, it has been reported that decreased lumbar and hamstring flexibility was attributed to LBP [35,40]. In addition, emerging evidence supports conservative treatment for improving hip mobility in non-specific LBP [7,41,42]. A meta-analysis study (2017) reported that restricted hamstring flexibility, as well as decreased lumbar motion and lordosis, were found to increase the risk of developing LBP [25]. On the other hand, Shakya et al. [43], Mistry et al. [44], and Stutchfield and Coleman (2006) [45] reported no association between LBP and hamstring tightness among students. A recent systematic review (2019) also documented insufficient evidence to support an association between limited hip ROM and non-specific LBP [26]. The authors of the latter suggested viewing this conclusion cautiously due to the low quality of their supportive evidence. We have attributed this discrepancy to the following reasons: (1) the diversity of the muscle flexibility measurements, (2) the lack of a border limit to discriminate between a normal and extreme range of flexibility/lengthening, and (3) the assortment of the study sample (e.g., students, athletes). Therefore, one could assume that both increased and decreased hamstring flexibility at the extremes of the range could alter the kinematics around the lumbopelvic girdle leading to LBP. We also suggest that clinicians should establish a normal range of hamstring length and be aware when they perform stretching for tight hamstrings in participants with LBP. 

Our results rule out any relationship between the length of iliopsoas and LBP. Supporting evidence was also obtained from the study of Volpato et al. [46] and Nourbakhsh et al. [14].

The association between study-related stress and LBP has been well documented in other cohort studies on healthcare and general populations [24,47,48,49]. For example, a systematic review and meta-analysis study confirmed that biopsychosocial factors (e.g., anxiety and mental pressure) were strongly related to LBP among nursing and medical students [48]. Recent evidence also indicates that different stress types should be taken into account when designing prevention and intervention programs for LBP [50,51]. We believe that academic institutions may consider developing and implementing proper strategies to mitigate the risk factors in these students.

When comparing muscle flexibility and gender in participants of the same age, it is apparent from our data and others [43,44,52,53,54,55] that females are more flexible than males. Notably, the effect of gender on joint ROM and muscle length was considered joint and motion-specific [56].

The current study also revealed a low prevalence of hip muscle tightness (20% for the hamstrings and 4% for iliopsoas) that contradicts others [43,57]. One study has reported a medium prevalence of hamstring tightness (40.19%) among physiotherapy students in Nepal [43]. Thakur and Rose (2020) [57] examined 80 healthy college students (40 males and 40 females) and showed a great prevalence (90% to 96%) of hamstring tightness (severe to tight) among males and females. Hip muscle tightness can be caused due to sustained sedentary behavior/and or inactive individuals. Nevertheless, all participants in the current study are in their first year of academic education (contrary to other studies) and we assume that a longer time is needed to detect the influence of sedentary behavior on muscle flexibility. A high percentage (46%) of the students were involved in anaerobic activity, which positively correlated with muscle flexibility. In addition, the diversity of the methodology for measuring muscle lengthening may affect the outcomes. 

Regarding muscle length symmetry, our data agree with that of others who reported no significant differences between muscle flexibility of the right and left extremities [58,59,60,61], but challenge the study of Stutchfield and Coleman [45].

Limitations of the study. This study involved a small sample size from a single academic centre. The self-reported questionnaire could include some biases (e.g., recall and socioeconomic factors). The duration of pain (e.g., acute or chronic) as well as pain location (e.g., right and left) were not described, and an unequal ratio of males and females was reported.

## 5. Conclusions

The current study indicates that the majority of first-year undergraduates manifest normal hip muscle lengthening (hamstrings—80%, iliopsoas—96%). Muscle length is gender-dependent, lacking significant differences between the right and left sides. Increased hamstring flexibility and stress-related study were considered predictive factors for LBP. We also believe that further research with a greater sample size is needed to shed light on the normal and extreme ranges of hamstring muscle length as well as to reproduce the association between increased muscle length and LBP. 

## Figures and Tables

**Table 1:** Sample size characteristics.

	n/(%) or Mean ± SD
Male	13 (19)
Female	57 (81)
Mean age (year)	25 ± 6
Mean BMI (kg/m^2^)	23.7 ± 4
Right hand dominant	63 (90)
Marital status:	
Single	55 (79)
Others	15 (21)
Smoking	8 (11)
General chronic diseases	19 (27)
Constant medication use	12 (17)
Religion & faith:	
Secular	41 (59)
Traditional	10 (14)
Religion and orthodox	15 (21)
Others	4 (6)
Involved with aerobic physical activities (e.g., walking and swimming)	35 (50)
Involved with anaerobic exercise (e.g., pilates and yoga)	32 (46)
Sustained daily sitting:	
Up to 3 h	36 (51)
>3 to 5 h	23 (33)
>5 h	11 (16)
Total daily sitting:	
Up to 6 h	31 (44)
Between 6 and 8 h	33 (47)
>8 h	6 (9)
Study-related stress:	
Very high–quite high	39 (56)
Little–none	31 (44)
Low back pain in the last week	32 (46)
Hamstring flexibility (degree) right	92.2 ± 17
Hamstring flexibility (degree) left	91.8 ± 17
Hamstring shortness	14 (20)
Iliopsoas flexibility (degree) right	11.6 ± 8
Iliopsoas flexibility (degree) left	12.4 ± 7
Iliopsoas shortness	3 (4)

**Table 2:** Mean muscle lengthening (right and left) by sex and physical activities.

Muscle Lengthening (Degree ± SD)	Sex		Aerobic Activity		Anaerobic Activity	
	Males (n = 13)	Females (n = 57)	Yes (n = 35)	NO (n = 35)	Yes (n = 32)	NO (n = 38)
Right hamstrings	78.5 ± 13 vs. 95.2 ± 17 *p* = 0.001		91.4 ± 18 vs. 92.8 ± 16 *p* = 0.736		97.7 ± 17 vs. 87.4 ± 16 *p* = 0.015	
Left hamstrings	80.6 ± 10 vs. 94.4 ± 17 *p* = 0.001		91.6 ± 16 vs. 92 ± 18 *p* = 0.915		97.6 ± 18 vs. 87 ± 15 *p* = 0.013	
Right iliopsoas	5.7 ± 8 vs. 12.8 ± 7 *p* = 0.012		11.2 ± 10 vs. 11.9 ± 6 *p* = 0.719		14.4 ± 8 vs. 9.1 ± 7 *p* = 0.007	
Left iliopsoas	7.6 ± 8 vs. 13.5 ± 7 *p* = 0.031		12.2 ± 9 vs. 12.6 ± 6 *p* = 0.818		15.5 ± 7 vs. 9.7 ± 6 *p* = 0.002	

**Table 3:** Muscle lengthening and low back pain.

Muscle Lengthening	With Low Back Pain (n = 32)	Without Low Back Pain (n = 38)
Right hamstrings (degree ± SD)	97 ± 16 88 ± 17 *p* = 0.029	
Left hamstrings (degree ± SD)	96.5 ± 19 87.8 ± 15 *p* = 0.042	
Right iliopsoas (degree ± SD)	13.5 ± 8 9.8 ± 8 *p* = 0.066	
Left iliopsoas (degree ± SD)	14 ± 7 11 ± 8 *p* = 0.115	

**Table 4:** A logistic regression analysis for the variables associated with LBP among first-year students.

Variable	OR	95% CI	*p* Value
BMI	0.893	0.784–1.018	0.090
Study-related stress	3.836	1.330–11.063	0.013
Hamstring length Rt.	1.035	1.003–1.069	0.033

OR—odds ratios, CI—confidence intervals, BMI—body mass index, Rt—right, LBP—low back pain.

## Data Availability

Datasets are available to download on request. Requests should be directed to the corresponding author.
